# A longitudinal, multi-parametric functional MRI study to determine age-related changes in the rodent brain

**DOI:** 10.1016/j.neuroimage.2020.116976

**Published:** 2020-09

**Authors:** Andrew Crofts, Melissa Trotman-Lucas, Justyna Janus, Michael Kelly, Claire L. Gibson

**Affiliations:** a Department of Neuroscience, Psychology & Behaviour, University of Leicester, Leicester, UK; b School of Psychology, University of Nottingham, Nottingham, UK; c Preclinical Imaging Facility, Core Biotechnology Services, University of Leicester, Leicester, UK

**Keywords:** Aging, fMRI, fMRS, Imaging, Preclinical models, ASL, Arterial spin labelling, BOLD, blood oxygen level dependent, CBF, cerebral blood flow, TE, echo time, FID-A, FID-applications toolkit, FEAT, FMRI expert analysis tool, FSL, FMRIB software library, FAST, FMRIB’s automated segmentation tool, FID, free induction decay, fMRI, functional magnetic resonance imaging, fMRS, functional magnetic resonance spectroscopy, LASER, localisation by adiabatic selective refocusing, MRI, magnetic resonance imaging, MRS, magnetic resonance spectroscopy, MCFLIRT, motion correction with FMRIB’s linear registration tool, MELODIC, multivariate exploratory linear optimised decomposition into independent components, NAA, N-acetyl aspartate, NMR, nuclear magnetic resonance, OIS, optical imaging spectroscopy, FMRIB, Oxford Centre for Functional MRI of the Brain, PET, positron emission tomography, S1FL, primary somatosensory cortex (forepaw region), rBET, rat brain extraction tool, TR, repetition time, TARQUIN, totally automatic robust quantification in NMR

## Abstract

As the population ages, the incidence of age-related neurological diseases and cognitive decline increases. To further understand disease-related changes in brain function it is advantageous to examine brain activity changes in healthy aging rodent models to permit mechanistic investigation. Here, we examine the suitability, in rodents, of using a novel, minimally invasive anaesthesia protocol in combination with a functional MRI protocol to assess alterations in neuronal activity due to physiological aging. 11 Wistar Han female rats were studied at 7, 9, 12, 15 and 18 months of age. Under an intravenous infusion of propofol, animals underwent functional magnetic resonance imaging (fMRI) and functional magnetic resonance spectroscopy (fMRS) with forepaw stimulation to quantify neurotransmitter activity, and resting cerebral blood flow (CBF) quantification using arterial spin labelling (ASL) to study changes in neurovascular coupling over time. Animals showed a significant decrease in size of the active region with age (P ​< ​0.05). fMRS results showed a significant decrease in glutamate change with stimulation (ΔGlu) with age (P ​< ​0.05), and ΔGlu became negative from 12 months onwards. Global CBF remained constant for the duration of the study. This study shows age related changes in the blood oxygen level dependent (BOLD) response in rodents that correlate with those seen in humans. The results also suggest that a reduction in synaptic glutamate turnover with age may underlie the reduction in the BOLD response, while CBF is preserved.

## Highlights

• Describe a novel anaesthetic protocol to examine age-related alterations in neuronal activity in rodents.
• Size of the BOLD signal in the somatosensory cortex decreased with age.
• Reduction in glutamate turnover with age.
• No change in resting CBF with age.

## 1 Introduction

Functional MRI (fMRI) is a non-invasive imaging method ideally suited to longitudinal studies and is an important tool in the study of healthy aging and age-related neurological disease. In humans the haemodynamic response detected by fMRI becomes less efficient with age (D’Esposito et al., 1999; Huettel et al., 2001), and many progressive age-related diseases such as hypertension and Alzheimer’s disease are characterised by a breakdown in neurovascular coupling (Stanimirovic and Friedman, 2012) leading to hypoxia, neuronal loss, and cognitive decline (Girouard and Iadecola, 2006). Thus, studying healthy aging may provide insight into risk factors for age-related disease. While many studies exist of healthy aging using fMRI in humans, to our knowledge no studies exist at this time examining healthy aging using fMRI in rodent models. Understanding healthy aging in rodent models will allow changes associated with normal development and disease-related changes to be identified in rodent models of disease, improving studies testing therapeutics and/or the understanding of disease progression in controlled conditions.

Current preclinical fMRI methods are not optimised for longitudinal studies (Crofts et al., 2019). In order to perform MRI on rodents, most studies use anaesthesia to minimise movement and distress to the animal (Tremoleda et al., 2012). Although MRI of awake animals is possible, there are many practical challenges to overcome (King et al., 2005; Lahti et al., 1999; Martin et al., 2013). Many previous fMRI studies use injectable anaesthetics, such as urethane or alpha-chloralose, with toxic, carcinogenic or harmful properties (Dijkhuizen et al., 2001; Kennerly et al., 2005) and therefore can only be used in non-recovery studies (Tremoleda et al., 2012). Inhaled anaesthetics, such as isoflurane or halothane, suppress the BOLD signal and shift the balance between capillary and arterial BOLD signal, masking long term changes in activity and artificially increasing the effect of noise or small variations (Paasonen et al., 2018; Schroeter et al., 2014; Tsurugizawa et al., 2016). Medetomidine may be a viable candidate for use in preclinical longitudinal studies, but the variable depth of anaesthesia can cause complications (Tremoleda et al., 2012; Weber et al., 2006). Propofol, an injectable anaesthetic common in veterinary practice is used here. Propofol demonstrates a less pronounced effect on CBF, BOLD signal or the haemodynamic response function when compared to other anaesthetics (Griffin et al., 2010; Kelly et al., 2010; Paasonen et al., 2018; Schroeter et al., 2014; Tremoleda et al., 2012; Van Zijl et al., 2012). Propofol is also known to be rapidly metabolised and removed from the body, and has previously been shown to have no cumulative effects from repeated use, making it suitable for long term studies (Tremoleda et al., 2012). While propofol has been shown to have adverse effects on the blood brain barrier in juvenile mice (Sharma et al., 2014), studies of aged rats have shown no adverse effects of propofol on the blood brain barrier (Hu et al., 2014), and so this is not thought to be a risk in adult animals. In animals anaesthetised with propofol, the haemodynamic response function has also been shown to more closely match that of awake animals than other anaesthetics (Schlegel et al., 2015). This has also been shown in mice anaesthetised with a fentanyl/isoflurane mixture, with a similar signal amplitude to animals recieving propofol (Sharp et al., 2015), suggesting that Fentanyl may be an alternative to propofol for longitudinal studies. The main disadvantages of Fentanyl are the possibility of prolonged recovery time (>4 ​h) and possibility of hypotension, which may pose a welfare risk in older animals (Tremoleda et al., 2012).

Changes in the human brain with aging have been well characterised with task-based and resting-state fMRI. The most notable change in task-based BOLD signal with aging is a significant reduction in the number of active voxels, 40–50% lower than observed in young subjects (Huettel et al., 2001; Kannurpati et al., 2010; West et al., 2019). Between-subject variability is greater in an aged cohort than a young cohort, possibly due to the cumulative effects of lifestyle factors over time (D’Esposito et al., 1999; Kannupati et al., 2010). This reduction in the size of the activated region may be due to a combination of vascular and neuronal changes and their contribution to the overall BOLD signal change may vary depending on the region of interest. When vascular changes are considered, reduced BOLD signal in motor regions is found to be largely attributable to vascular alterations, while in cognitive tasks BOLD signal reduction is mainly attributable to neuronal loss (Kannurpati et al., 2010). This illustrates an important limitation of BOLD fMRI that should be accounted for – while changes in activity can be imaged, other experiments are needed to determine the relative contributions of vascular and neuronal factors and the processes behind these changes. Understanding the causal relationship between vascular and neuronal changes with age is important in an ageing population in order to better treat age-related cognitive decline, target risk factors for neurodegenerative disease, and monitor rehabilitation in patients suffering from age-related brain injury. Understanding changes with healthy aging in preclinical models is also important, in order to improve on models of age-related disease.

In order to understand the neuronal factors behind the BOLD signal, a non-invasive method of quantifying neuronal activity is required. Electrophysiology methods are invasive and are therefore not practical for longitudinal studies due to their impact on animal welfare. A method that has previously been suggested to give a non-invasive measurement of neuronal activity is functional MR spectroscopy (fMRS). Obtaining two spectra, with and without stimulation, and subtracting the two, the difference in relative concentrations of the neurotransmitter glutamate can be detected and used to infer neuronal activity in the region (Gussew et al., 2010). This method is limited in spatial resolution, measuring a voxel several millimetres wide rather than the sub-millimetre resolution provided by preclinical fMRI, however this may be useful when paired with fMRI in studies of a single, known region of interest (Stanley and Raz, 2018). In this study, we used fMRI to explore age-related changes in somatosensory cortex activity in response to forepaw stimulation. The stimulus design used, which applies a current of approximately 2.3 ​mA, has been previously shown to only activate somatosensory fibres, and not pain related fibres, while providing a robust BOLD and CBF signal under propofol anaesthesia (Griffin et al., 2010). Understanding age-related changes in healthy animals using fMRI is important in improving the current understanding of age-related cognitive decline and age-related pathologies. Additional fMRI methods can improve the understanding of how neuronal and vascular components contribute to what is observed with BOLD fMRI.

## 2 Materials and methods

### 2.1 Animals

This study was conducted in accordance with the UK Animals (Scientific Procedures) Act, 1986 and following institutional ethical approval by the University of Leicester Animal and Welfare Ethical Review Body. All experiments are reported in accordance with the Animal research reporting of in vivo experiments (ARRIVE) guidelines (Kilkenny et al., 2010). A total of 11 female rats (Wistar Han, code CRL:WI(Han), Charles River Laboratories) were used in the current study; all animals completed the study. Rats aged 3 months on arrival were group housed in two groups of four animals, and one group of three animals, in double decker cages (floor area 1862 cm^2^, height 38 ​cm, Techniplast, UK) and given daily access to a playpen for 2–6 ​h. Animals were fed PMI 5LF2 diet (Lab Supply, Fort Worth, Texas, USA) ad libitum and mains water that had been UV treated in house (Severn Trent, UK). Animals were first acclimatised to single level playpens in groups of 3/4 for one week, followed by two weeks of mixing up to 8 animals from two different cages together, then mixing all animals into one playpen. At 12 months of age, the playpen was replaced with a three-storey chinchilla cage kept in a ventilator cabinet, maintained at room temperature, with an isolated air supply and red glass doors to block external light sources. Preventing outside air and external light prevents animals from seeing or smelling humans while in the playpen, with the intention of promoting more natural behaviour without anxiety from potential threats.

### 2.2 Experimental schedule

Bench experiments were performed on animals at 5 months old to test the anaesthesia protocol and identify the correct dose rate. The protocol described below was performed in order to test the initial dose rates of 7.5 ​mg/kg bolus followed by 42 ​mg/kg/hr infusion of propofol (Griffin et al., 2010), without MRI scanning. When this did not induce a sufficient depth of anaesthesia in initial tests, with animals displaying motion after isoflurane was discontinued, animals were returned to isoflurane before increasing the dose rate. Dose rates were increased in 10% increments until a dose rate of 9 ​mg/kg bolus followed by 54 ​mg/kg/hr infusion was identified as the lowest dose required to maintain anaesthesia, defined as the animal showing no motion or change in breathing rate once isoflurane had been discontinued, and all animals remained stable using this dose.

Animals were weighed prior to all MRI scans, and weighed daily for two days after MRI scans to identify any weight loss related to anaesthesia. MRI scans were undertaken at the following time points; 7, 9, 12, 15 and 18 months. At 18 months old, immediately following the final MRI scan, animals were humanely killed while under propofol anaesthesia. 6 animals were sacrificed using intravenous pentobarbitone. 5 animals were sacrificed using intraperitoneal pentobarbitone, and transcardially perfused with formaldehyde solution.

### 2.3 Anaesthesia protocol

Animals were anaesthetised with isoflurane (5% induction; 2–3% maintenance) in 100% oxygen. Tail vein cannulation was performed before transferring the animals to the MRI bed. A bolus of 9 mg/kg/min propofol was administered for 1 ​min using a syringe driver (Harvard Apparatus, Cambridge, Massachusetts, USA) and the isoflurane was gradually reduced to 0%, with animals still breathing 100% oxygen. Respiration was monitored using a respiration pillow (Small Animal Instruments Inc. Stony Brook, New York, USA), temperature monitored using a rectal probe and maintained using a fan heater activated when measured temperature dropped below 37 ​°C (Small Animal Instruments Inc. Stony Brook, New York, USA), and heart rate and blood oxygen saturation monitored using a pulse oximeter (Starr Life Sciences, Oakmont, Pennsylvania, USA). Copper electrodes were inserted subcutaneously into the dorsal surface of the right forepaw between digits 1–2 and 2–3 (Hyder et al., 1994). Three minutes after the bolus ended, a continuous infusion of propofol was given at 54 ​mg/kg/hr for the duration of the scan. Following the scan, animals were allowed to recover in a separate cage. Analgesic cream (EMLA cream, AstraZeneca, UK) was applied to the forepaw where electrodes had been removed, and animals were provided with water and wet food pellets to prevent dehydration after anaesthesia. Animals were awake within 15 ​min following removal of anaesthesia, and moving freely within 30 ​min, at which point they were returned to their home cage.

### 2.4 MRI protocol

MRI scans were performed on a 9.4T Small Animal MRI scanner (Agilent Technologies), using a 72 ​mm RF volume transmit coil and a 2-channel surface receive coil (Rapid Biomedical, Rimpar, Germany). Coronal scout images were used to locate bregma, and three 1.5 ​mm slices were selected with bregma in the middle slice. A shimming voxel with dimensions 10 x 9 ​× ​4.5 ​mm was positioned to cover the centre of all three slices, excluding non-brain tissue or tissue outside the slices of interest. Fast, automatic shimming technique by mapping along projections (FASTMAP, Gruetter, 1992) was used to shim these slices to a 50% water linewidth between 20 and 35Hz. Rats were switched from breathing oxygen to room air, pumped at 2 ​L/min using an air pump (Wiz-Air, Clarke Tools, Dunstable, UK). fMRI was performed for 9 ​min using a gradient echo EPI sequence (TR ​= ​250 ​ms, TE ​= ​22 ​ms, kzero ​= ​8, shots ​= ​2, data matrix ​= ​128 x 128). The forepaw was stimulated at 10 ​mV, 10Hz, pulse width 1μs, with a block design of 60s off, 30s on. Following this, a 4 x 4 ​× ​4 ​mm voxel was positioned over the left somatosensory cortex at bregma, and manual shimming was performed to a 50% water linewidth of <25Hz. A localisation by adiabatic selective refocusing (LASER, Slotboom et al., 1991) MRS sequence (TR ​= ​2000 ​ms, TE ​= ​14.54 ​ms, 270 arrayed averages) was used to perform fMRS over 9 ​min, using the same forepaw stimulation paradigm used for fMRI. For analysis, the first 30 ​s of each “off” block were discarded to allow time for metabolites to return to baseline levels. Oxygen saturation was maintained above 80% during functional experiments (Tremoleda et al., 2012). Animals were switched back to 100% oxygen after all functional scans were complete, in order to minimise any welfare risks extended use of anaesthesia and room air. After being returned to 100% oxygen, a time-of-flight (TOF) angiography scan was performed in order to position the labelling plane for ASL, followed by a T2 weighted structural scan. Using the TOF angiography image, the carotid artery was located and a labelling plane placed with a gap of −17mm from bregma. 6 ​min of continuous arterial spin labelling (CASL) was performed on a single 1.5 ​mm slice placed over bregma (TI ​= ​1500 ​ms, TR ​= ​2500 ​ms, TE ​= ​10 ​ms, shots ​= ​1, kzero ​= ​16, data matrix ​= ​128 x 128).

### 2.5 Data analysis

A fMRI analysis pipeline was developed using FSL (www.fmrib.ox.ac.uk/fsl; Smith et al., 2004). MCFLIRT (Jenkinson et al., 2002), rBET (a modified version of BET to account for differences in the shape of the rat brain compared to the human brain, Smith, 2002; Wood et al., 2013), and FAST (Zhang et al., 2001) were used for motion correction, brain extraction and bias field correction respectively. Independent component analysis for artefact removal, using MELODIC (Beckmann and Smith, 2004) were performed prior to time-series analysis in FEAT (Woolrich et al., 2001) to visualise the BOLD response. Cluster analysis was performed on the first-level FEAT outputs to determine number of active voxels, maximum % signal change within the cluster, and mean % signal change across the cluster. In each scan, time to peak was calculated for each of the six stimulus blocks and averaged, with a temporal resolution of 0.5 ​s (Baumann et al., 2010).

fMRS spectra were separated into “off” and “on” blocks and averaged using the MATLAB FID-A toolkit (Simpson et al., 2017). The two spectra were analysed using the TARQUIN MRS analysis package (www.tarquin.sourceforge.net) to determine relative concentrations of glutamate. Metabolite concentrations from “off” blocks were subtracted from “on” blocks to determine the difference in glutamate concentration, and the difference in glutamate change (ΔGlu) was averaged across subjects. “Off” blocks were also used to quantify NAA and Inositol, used as markers of neuronal viability and inflammation, respectively.

Control and tag ASL images were first concentrated into a single image of two volumes. The Oxford ASL toolkit for FSL was used to subtract the tag image from the control image to create a difference image. FSLmaths was used to apply a modified Bloch equation (Williams et al., 1992) to the data in order to quantify CBF in absolute units. CBF map images were given an upper threshold of 5 to remove large outliers. This was determined based on the histogram of voxel intensities, in which some scans displayed a small number of single voxels with large intensities between 5 and 80 having a large influence on the mean. Non-zero mean and standard deviation CBF were calculated using FSLstats.

Statistical analysis was performed in Graphpad Prism (Version 8, California, USA). Data are shown as mean ​± ​standard deviation (SD). Changes over time were analysed using a repeated measures ANOVA, fitting a mixed effects model to account for missing values, and the criterion for statistical significance was P ​< ​0.05. Where statistically significant differences were found, post-hoc testing to compare individual time points was performed using the Tukey-Kramer multiple comparisons test.

## 3 Results

### 3.1 Animal body weight and physiological monitoring

**Fig. 1:**
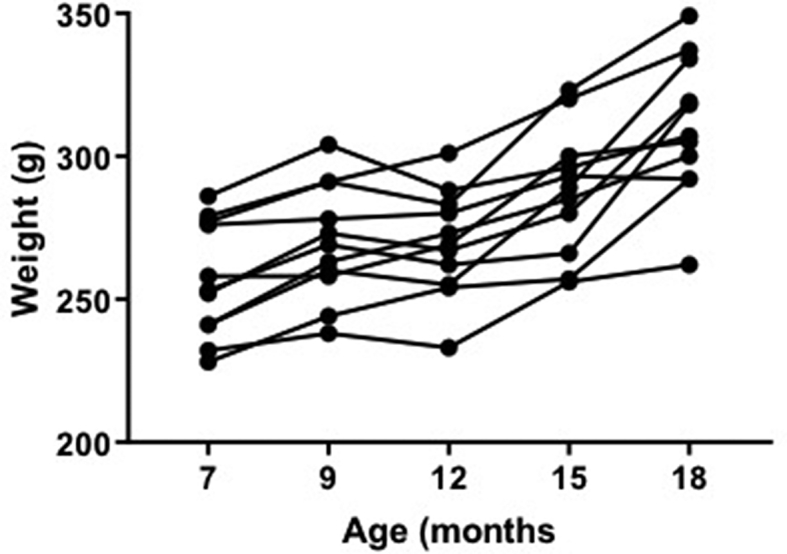
Individual rat weights at 7, 9, 12, 15 and 18 months (n ​= ​11).

**Fig. 2:**
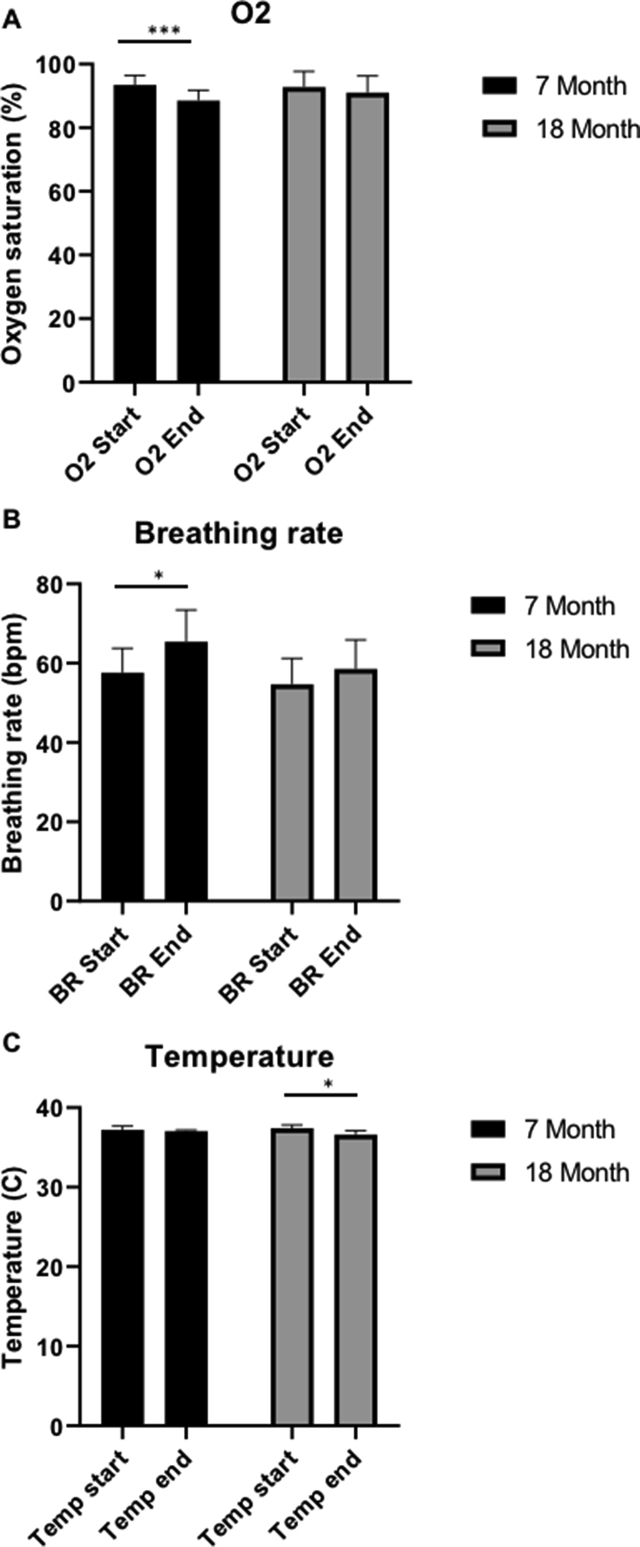
Oxygen saturation (A), Breathing rate (B) at the start and end of the period on room air, and Temperature (C) at the start and end of scanning, in 7 months old and 18 months old animals (∗P ​= ​0.05, ∗∗∗P ​= ​0.001). Data is displayed as mean ​± ​SD, n ​= ​8 and 7 respectively.

Body weight was found to significantly increase with age (Repeated measures ANOVA, F ​= ​14.19, P ​< ​0.0001), from 253 ​± ​20.26 ​g ​at 7 months old to 310 ​± ​24.61 ​g ​at 18 months, and remained within the expected weight range for healthy Wistar Han rats (Fig. 1). Post-hoc comparisons showed significant differences when comparing 7 months vs 9 months (P ​= ​0.0012), 7 vs 12 months (P ​= ​0.012), 7 vs 15 months (P ​= ​0.0002), 7 vs 18 months (P ​= ​0.0001), 9 vs 15 months (P ​= ​0.0196), 9 vs 18 months (P ​= ​0.0004), 12 vs 15 months (P ​= ​0.0047), 12 vs 18 months (P ​= ​0.0004), and 15 vs 18 months (P ​= ​0.012). During scanning, while breathing room air, oxygen saturation remained above 80%, which has been previously defined as safe (Tremoleda et al., 2012). In 7 month old animals, Oxygen saturation (Fig. 2A) was found to decrease from 93.48 ​± ​2.97% at the start of the period on room air, to 88.54 ​± ​3.22% at the end of the period on room air, approximately 35 ​min (paired *t*-test, P ​= ​0.0002). However, in 18-month-old animals, oxygen saturation did not significantly change from 92.83 ​± ​4.89% in this time (Paired *t*-test, P ​= ​0.0820). The breathing rate (Fig. 2B) while on room air also significantly increased between the start and end of this period in 7 month old rats, from 57.6 ​± ​6.18 bpm to 65.4 ​± ​8.04 bpm (paired *t*-test, P ​= ​0.0011), however at 18 months, breathing rate did not significantly change from 54.71 ​± ​6.55 bpm (paired *t*-test, P ​= ​0.1267). In 7 month old animals, temperature (Fig. 2C) did not change over time from 37.2 ​± ​0.47 ​°C (paired *t*-test, P ​= ​0.3629), however in 18 month old animals, temperature did significantly decrease during scans from 37.43 ​± ​0.377 ​°C to 36.63 ​± ​0.46 ​°C (paired *t*-test, P ​= ​0.0224).

### 3.2 Functional MRI

**Fig. 3:**
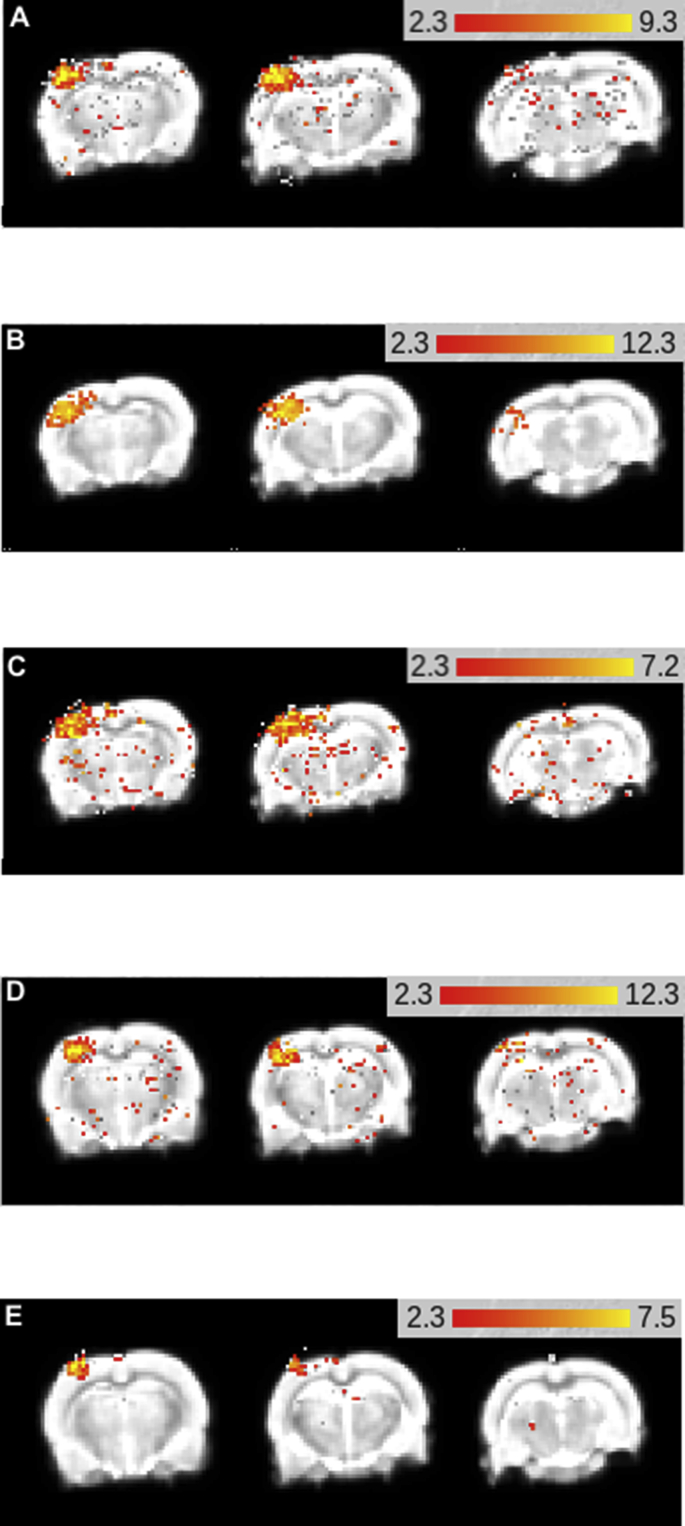
Example images displaying S1FL activation in a representative rat at 7 months (A), 9 months (B), 12 months (C), 15 months (D) and 18 months. All analysis was performed with a z threshold of 2.3 and cluster P threshold of 0.01.

**Fig. 4:**
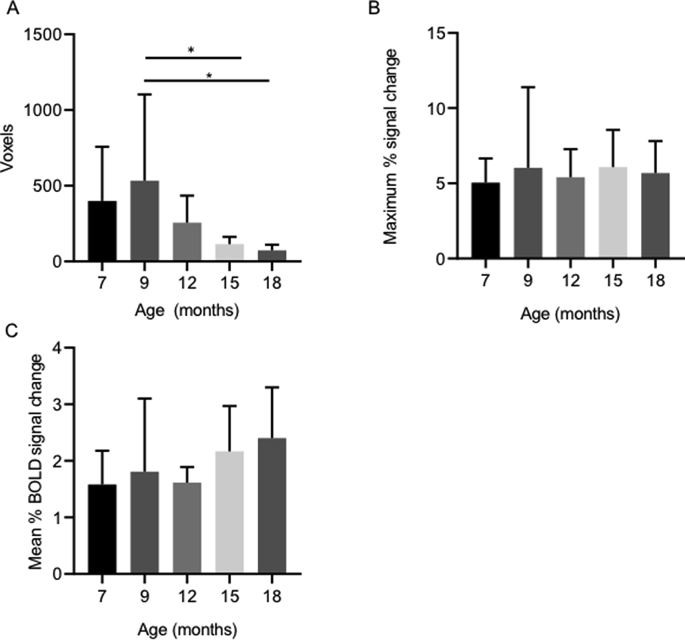
Effect of age on various aspects of the BOLD signal including the maximum BOLD signal change in S1FL (A), the mean BOLD signal change of all active voxels in S1FL (B), and the number of active voxels in S1FL (C). There was a significant decrease in the number of active voxels with age (∗P ​= ​0.05, between various times points). Data is displayed as mean ​± ​SD, n ​= ​8, 8, 8, 10, and 7 ​at 7, 9, 12, 15 and 18 months respectively.

**Fig. 5:**
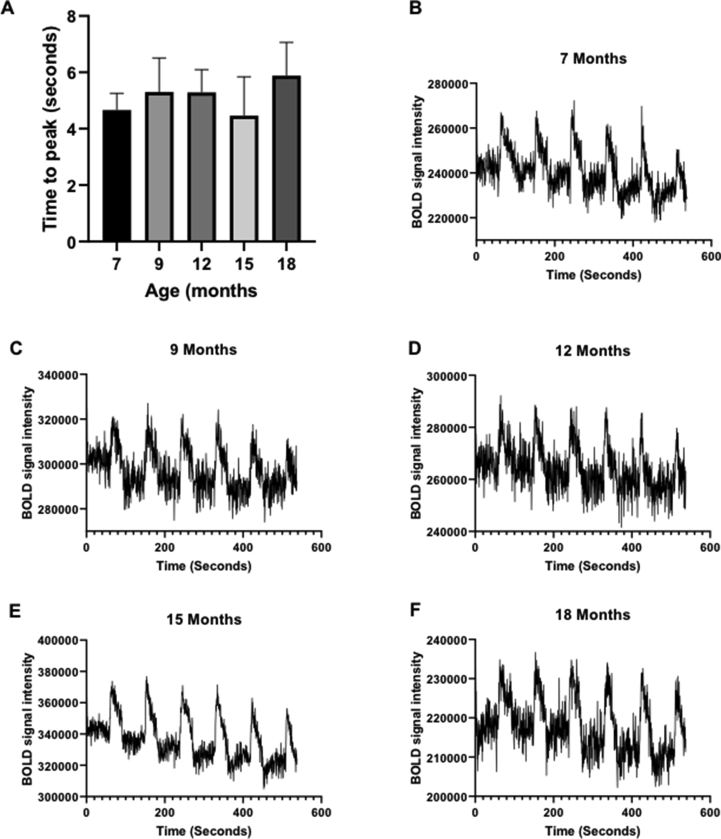
(A) Bar graph showing time to peak of BOLD signal at all time points. Data is displayed as mean ​± ​SD, n ​= ​8, 8, 8, 10, and 7 ​at 7, 9, 12, 15 and 18 months respectively. (B–F) Average time series plots of BOLD signal at each time point, taken from the voxel with the highest z statistic. n ​= ​8, 8, 8, 10, and 7 ​at 7, 9, 12, 15 and 18 months respectively.

First-level analysis of BOLD fMRI data was performed in FEAT (Fig. 3) and was used to quantify three aspects of the BOLD signal: number of active voxels in S1FL, maximum % signal change in S1FL, and mean % signal change in S1FL (Fig. 4). The number of active voxels was found to significantly decrease with age (F (4, 27) ​= ​3.727, P ​= ​0.0153), with the number decreasing from 399.5 ​± ​356.8 ​at 7 months to 76.7 ​± ​37.0 ​at 18 months old. Post-hoc testing found that there was a significant decrease in the number of active voxels between the ages of 9 months (532.8 ​± ​570.3) and 15 months (114.7 ​± ​46.91 ​m ​P ​= ​0.0367), and between 9 months and 18 months (76.7 ​± ​37, P ​= ​0.0286). Standard deviation was also shown to significantly decrease with age (Brown-Forsythe test, P ​= ​0.0001). No significant difference was found in the maximum BOLD signal change (F (4, 27) ​= ​0.1963, P ​= ​0.9381) or mean BOLD signal change (F (4, 36) ​= ​1.422, P ​= ​0.2465) with age. Time-to-peak of the BOLD signal was not found to significantly change with age (F (4, 27) ​= ​2.210, P ​= ​0.0946, Fig. 5).

### 3.3 Functional MRS

**Fig. 6:**
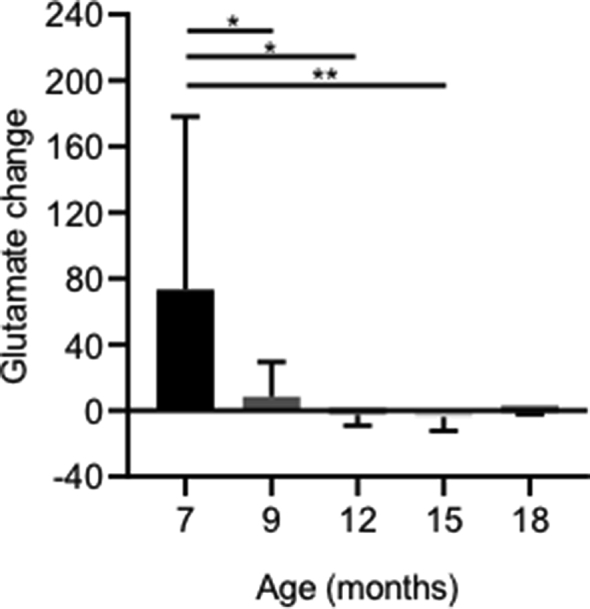
Effect of age on glutamate change in S1FL with forepaw stimulation. There was a significant decrease in the glutamate signal change with increasing age (∗P ​= ​0.05, between various times points). Data is displayed as mean ​± ​SD, n ​= ​8, 10, 9, 11, and 4 ​at 7, 9, 12, 15 and 18 months respectively.

**Fig. 7:**
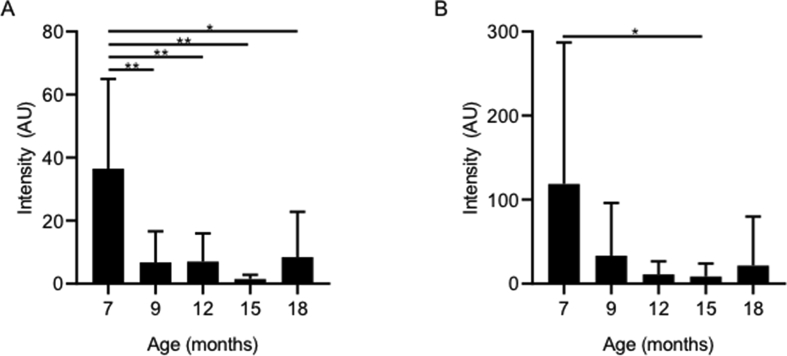
Effect of age on intensity of NAA (A) and Inositol (B) levels with age in the somatosensory cortex. Both NAA and Inositol levels significantly decreased with increasing age (∗P ​= ​0.05, between various times points). Data is displayed as mean ​± ​SD, n ​= ​8, 10, 9, 11, and 4 ​at 7, 9, 12, 15 and 18 months respectively.

The change in glutamate signal between off and on blocks (ΔGlu) decreased significantly (F (4, 37) ​= ​4.018, P ​= ​0.0083) from 7 months (73.4 ​± ​103.6AU) to 18 months (−1.08 ​± ​1.19AU). Post-hoc testing showed significant differences between 7 and 9 months (P ​= ​0.0447), 7 and 12 months (P ​= ​0.0162) and 7 and 15 months (P ​= ​0.009). From 12 months onwards, the glutamate signal change became negative, showing an overall decrease in glutamate with activation (Fig. 6). NAA and Inositol were also quantified (Fig. 7). Quantification of NAA displayed a significant decrease (F (4, 27) ​= ​5.267, P ​= ​0.0025) in NAA from 36.50 ​± ​28.46AU at 7 months to 8.41 ​± ​14.40AU at 18 months, with post-hoc testing showing significant decreases between 7 months and 9 months (P ​= ​0.0076), 12 months (P ​= ​0.0083), 15 months (P ​= ​0.0029) and 18 months (P ​= ​0.048). Inositol was found to significantly decrease (F (4,41) ​= ​1.68, P ​= ​0.0417) from 118.53 ​± ​168.3AU at 7 months to 21.7 ​± ​58.1AU at 18 months. 7 months and 15 months (8.63 ​± ​15.54AU) were found to be significantly different in post-hoc testing (P ​= ​0.036).

### 3.4 Arterial spin labelling

**Fig. 8:**
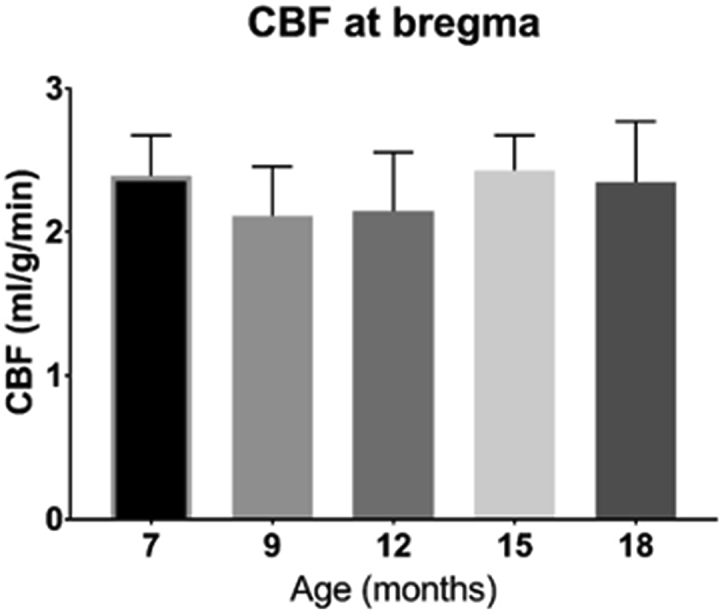
The effect of age on resting CBF at bregma. There were no significant differences in resting CBF at bregma at any of the time points measured. Data is displayed as mean ​± ​SD, n ​= ​11 ​at all time points apart from n ​= ​10 ​at 18 months.

Mean CBF across the slice did not change significantly between 7 and 18 months (F (4, 39) ​= ​1.438, P ​= ​0.2398), with low variability between subjects (Fig. 8). Mean CBF at all time points was within a standard deviation of the expected value of 2.33ml/g/min observed in some previous rodent experiments (Thomas et al., 2006).

## 4 Discussion

This study investigated changes in the BOLD signal, glutamate turnover and CBF with age in healthy rats using fMRI. Propofol was determined to be a suitable anaesthetic for longitudinal studies, causing no detrimental effects to animal health and allowing detection of a strong BOLD signal compared to observations using other anaesthetics (Schroeter et al., 2014). The stimulus paradigm allowed for detection of a strong BOLD signal. Animals did exhibit increased breathing rate due to arousal from the somatosensory stimulus, which does represent a limitation in this method due to the possibility of motion artefacts. The anaesthetic regime was well tolerated, with young animals showing a small decrease in oxygen saturation over time during scanning. Aged animals did not show a change in oxygen saturation during scanning, and all animals maintained oxygen saturation above the safe limit of 80% (Tremoleda et al., 2012). The size of the BOLD signal in the somatosensory cortex decreased with age. fMRS showed a reduction in ΔGlu (glutamate during stimulation – glutamate during rest) from 9 months onwards, with ΔGlu becoming negative at 12 months old. ASL showed that resting CBF remained constant for the duration of the study.

Here we report, for the first time, a decrease in the BOLD signal during aging in rodents, which is consistent with human task-based fMRI studies comparing young and aged groups, where the active region is significantly reduced in size during motor tasks (Ances et al., 2009; Huettel et al., 2001). The maximum and mean signal change remained constant over all time points, and was consistently located in the centre of the ROI, which is consistent with human studies where the BOLD signal amplitude remains consistent in healthy aged subjects while the spatial extent of the BOLD response is reduced (D’Esposito et al., 1999; Huettel et al., 2001). Time-to-peak BOLD signal also remained constant at all time points. While the reduction in size of the active region is consistent with human studies, the reduction in variability between subjects with age is not consistent with human studies (D’Esposito et al., 1999). Aged human subjects show a greater variability in task-related BOLD signal than young subjects (Kannurpati et al., 2010). Our data shows a reduction in variability between 12 and 18 months. In healthy volunteers, there is uncontrolled variability in lifestyle factors and medical history, whereas in our study, all animals, as is standard in preclinical studies, experience the same highly controlled environment. Careful control of lifestyle factors in this manner provides an excellent platform for future treatment studies involving longitudinal fMRI (and other analyses).

While these results show similarities to observations in humans, they do differ from observations in aged rats using optical image spectroscopy, in which the haemodynamic response is measured using visible light through a cranial window. With this method, aged rats (40 months) were shown to display a larger active region in response to forepaw stimulation than 4 or 24-month-old animals (Dubeau et al., 2011a). This could be due to variation between groups, as a longitudinal study design was not used. Alternatively, this could be due to differences between fMRI and optical imaging. The BOLD signal and neurovascular signals detected by optical imaging show high spatial correlation (Kennerly et al., 2005, 2012). However, the higher temporal resolution of optical imaging (Dubeau et al., 2011a), or differences in analysis methods, may mean that optical imaging is more sensitive to an effect of aging that could not be detected by fMRI.

Reduction in size of an active region may be due to synaptic plasticity, neuronal death, reduction of neurovascular signalling, or reduced vascular reactivity (Girouard and Iadecola, 2006). In normal learning and development, or during recovery following ischaemic stroke, task-related activity may shrink over time as connections are formed and become more efficient (Penhue and Doyon, 2005). While this is unlikely in an aging group of animals, animals in our study were provided novel stimuli as part of the animals’ enrichment, which is thought to be beneficial for somatosensory cortex function. Animal welfare is also an important concern in longitudinal studies, and animals’ health over their lifetime can have a major impact on study outcomes. For this reason, access to an enriched environment providing more space, sensory enrichment and socialisation than allowed by standard rat housing was provided in accordance with previously identified requirements (Hutchinson et al., 2005). Neuronal death, reduced neuronal activity, reduced neurovascular signalling and reduced neurovascular activity are all elements of age-related changes to the brain, with varying effects depending on overall health (Girouard and Iadecola, 2006). Thus, the reduction in size of the active region is likely an effect of healthy ageing, while the low variability is likely due to the beneficial effects of the enrichment protocol. Future studies could take into account other effects on the fMRI analysis. For example, in human studies, grey matter volume has been shown to decrease with age, which could affect the BOLD signal. Modelling the difference in grey vs white matter may be used to compensate for this by calculating partial volume effects. However, this requires longer scan times to optimise contrast in structural scans (Dukart and Berolino, 2014), which was not practical at this stage due to the need to minimise scan time. A limitation of BOLD fMRI is that the BOLD signal is not a direct measure of neuronal activity. Impairment of neurovascular coupling or changes in vascular reactivity can confound interpretation of the BOLD signal. For this reason, a more direct measure of neuronal activity can be beneficial. Glutamate is the most common neurotransmitter in the brain (Walls et al., 2015), and so direct quantification of glutamate turnover may complement BOLD fMRI data when investigating neurovascular coupling. MRS is the least invasive method of measuring neurotransmitter levels in vivo, and though there are limitations with respect to spatial and temporal resolution, this method has previously been applied to quantify task- or stimulus-based changes in glutamate and lactate (Gussew et al., 2010; Stanley and Raz, 2018).

Here, we describe age-related changes in glutamate levels becoming negative from 12 months onwards. The changes observed in glutamate were seen earlier than changes observed in the BOLD signal. Human studies using MRS have shown a decrease in overall glutamate concentration at a relatively young age, with one study showing a reduction of 5 ​mmol/L between 20 and 55, and one study showing a similar change between 18 and 32 (Hadel et al., 2013; Marsman et al., 2013). The changes observed here with functional MRS may be due to decreasing efficiency of glutamate turnover mechanisms with age. Glutamate is normally stored in vesicles and released with activation. As glutamate is released, increased glutamate production occurs to refill the vesicles and glutamate is recycled with sustained activity through the glutamine-glutamate cycle (Walls et al., 2015). If this cycle is impaired with age, and glutamate production does not exceed or keep up with glutamate release, this may be measured as a net decrease in glutamate. If this is the case, it can also explain the reduction in BOLD signal observed with age. Glutamatergic receptors on astrocytes may be an important mediator of neurovascular coupling (MacVicar and Newman, 2015), and reduced glutamate turnover would lead to reduced glutamate diffusion outside the synaptic cleft. It is important to consider other mechanisms of neurovascular coupling however, as there is conflicting evidence regarding the role of metabotropic glutamate receptor 5 (mGluR5) on astrocytes in rats (Calcinaghi et al., 2011), and the presence of mGluR5 in the brains of adult mice (Sun et al., 2013). The range of metabolites detectable by MRS is more limited than other methods, such as PET, and so alternative, more invasive methods may be needed to study other neurovascular signalling pathways. Additionally, due to the low SNR of fMRS at the scan time used in this study, caution must be exercised in interpreting the findings. Further studies using longer scan times, or alternative MRS techniques to improve sensitivity or SNR are required to confirm this hypothesis. While the LASER sequence allows for a short TE, improving sensitivity to glutamate, techniques such as PRESS offer an improved SNR at the cost of a longer TE, which may be beneficial in the shorter acquisition times required for a stimulus-based experiment (Zhu and Barker, 2011). MEGA-PRESS (Mescher et al., 1998) or Carbon-13 spectroscopy (Lai et al., 2017) may also be useful, as sequences selective to glutamate can be designed, however these come at the cost of a reduced resolution or requirement for radiolabelled glutamate, respectively. Improving the sensitivity of the fMRS method this way is important for this method to be of value in understanding glutamate turnover in future studies, and to better understand the change in glutamate concentration seen in humans.

We also measured changes in the levels of the metabolites, NAA and inositol, as they are correlated with age-related cognitive decline in humans. NAA is reported to be a neuronal marker and an indicator of neuronal integrity as others have reported a correlation between NAA and neuronal density in patients with Alzheimer’s disease (Cheng et al., 2002). With respect to aging others have demonstrated a decrease in NAA levels in human studies (Boumezbeur et al., 2009; Lim and Spielman, 1997; Schmitz et al., 2018) which is consistent with the age-related decline in NAA we observed here and may be indicative of reduced neuronal density or neuronal function. However, changes in NAA levels in humans may be regionally dependent (Eylers et al., 2016) which we did not assess here, and further studies could correlate changes in such levels with alterations in cognitive function. Inositol, or myo-inositol, is considered to be a putative glial marker (Martin, 2007) and higher levels are seen in patients with, for example, Alzheimer’s disease or frontotemporal dementia, which might be expected in conditions associated with higher gliosis (Ernst et al., 1997). However, it is still not clear what changes in absolute levels of inositol represent – it may be that changes, as reported here, reflect changes in glial metabolism (Marajanska et al., 2017). The decrease in NAA and glutamate turnover after 7 months may suggest that this is the point where rodent brains mature from young adulthood to middle age, as some human studies have shown a decrease in NAA between at this time as formation of new pathways slows down (Cichoka and Beres, 2018), however, further study in humans and rodents is needed to fully understand such changes.

ASL, as used here, allows imaging of cerebral perfusion. No changes were seen in mean CBF across the lifespan of the animals studied here, and values were similar to those reported in previous rodent studies (Thomas et al., 2006), however some studies do report a lower value (Wegener et al., 2007) which may be due to variation between strains. This would suggest that mechanisms of cerebral autoregulation remained intact in these animals. Human studies vary in whether aging affects resting perfusion, showing either no change in CBF (Meltzer et al., 2000) or a decrease in CBF with age (Tarumi and Zhang, 2018). In this study, the consistent level of perfusion taken with the change in glutamate suggests that changes in the BOLD signal are caused by neuronal factors rather than vascular factors.

To our knowledge, this study for the first time shows age-related changes in stimulus-induced BOLD signal in a longitudinal study of rats. Through use of a novel anaesthesia protocol, which overcomes the limitations of other anaesthetics, a large active region was detected in the somatosensory cortex of young rats, which decreased in size over the animals’ lifetime. This is consistent with the difference in BOLD signal between young and old human volunteers. In contrast to human studies, the aged animals showed very low between-subject variability, which may be due to the animal cohort all experiencing the same living environment, while lifestyles in human volunteers are variable. A further study with a larger cohort may allow the age at which changes to the BOLD signal begin to be identified. There are some methodological issues in the current study to consider in terms of the short TR used, which, combined with limitations in shimming an adequate volume, did limit scans to three slices over the region of interest without loss of image quality. While this allowed for a high temporal resolution and a large number of repeats, whilst keeping scan time to 9 ​min, the limited field of view did prevent image registration to high-resolution T2 weighted images or to a standard space. Because of this, it was necessary to analyse images at first level, then number of active voxels and signal intensity recorded, and then group level comparisons were performed manually. Whole-brain fMRI would facilitate registration of images to a standard structural space, thus allowing voxel-wise group level analysis to be performed. As we have demonstrated the utility of the anaesthesia protocol, future studies can incorporate longer scan times, and maintain animals on room air for longer, and so whole brain images can be obtained either through improved shimming or a longer TR and additional stimulus blocks. fMRS also suffered from low SNR due to the need for a short scan time, as the total length of the protocol was kept to approximately 1 ​h to minimise risk from the novel anaesthetic protocol, which may be improved on in future studies. The issue of variable number of subjects at different time points can also be addressed. In this study, technical difficulties with scanner hardware and monitoring equipment did mean that some animals had to be recovered before completing the protocol, and additional scans to rectify this were avoided for welfare purposes. As no harmful effects of repeated scanning were observed here, future studies can use additional scans where necessary to ensure the number of animals studied is consistent at all time points. High variability at early time points is also a limitation of this study, and although use of a repeated measures statistical analysis may account for this in part, future studies must address this in the methodology. This may be a physiological factor in young adult rats, in which case a larger cohort would be necessary. Alternatively, if this is a result of hardware variability, further refinement of shimming protocols may be beneficial to minimise variation between scanning sessions.

To compare findings in animals and findings in humans, it is also important to consider the effect of anaesthetic. All anaesthetic agents cause a reduction in BOLD signal intensity and electrophysiological activity (Lahti et al., 1999; Schlegel et al., 2015). However, whether this effect is consistent across age groups, or becomes more or less pronounced with age has not yet been studied. To study this in a longitudinal experiment would require lengthy acclimatisation procedures to ensure animals remain still and are minimally distressed by the scanner, and knowledge of whether this acclimatisation is disrupted as animals age (Crofts et al., 2020). Until this can be made feasible, potential changes in the effect of anaesthetic with age are one limitation of these studies.

Wistar Han rats have been previously used as a model for aging (Korhonen et al., 1997; Badowska-Szalewska et al., 2017), as these animals show low tumour incidence and lower weight gain than other strains (Weber, 2017). The 2 year survival rate of Wistar Hans rats is 63% (Charles River, 2011), and so for welfare purposes, 18 months was determined to be a humane end point for this study. To equate this to human age, one month of aging in rats can be roughly equated to three human years (Sengupta, 2013), equating 18 months in rodents to 54 years of age in humans. Spectroscopy studies in humans have shown age-related changes in glutamate concentration before middle age (Hadel et al., 2013; Marsman et al., 2013), and changes in BOLD signal in humans have been shown between groups with mean ages of 24 and 58 (Kannurpati et al., 2010). Thus, 18 months can be considered old enough to display equivalent age-related changes in rats, while young enough to minimise the risk of illness. 7 months was determined to be an appropriate starting point, equating to 21 years of age in humans, and previous studies suggest that 5–6 months is the age at which rats can be considered socially mature (Sengupta, 2012). As this strain has been shown to remain healthy with the enrichment described, and exhibited no adverse effects from anaesthesia, future studies may extend the end point further, to observe changes in rats in the equivalent of 60s, 70s or 80s in human age. Studies into the effect of different levels of enrichment would also be beneficial, both to animal welfare and to understanding how lifestyle affects brain ageing. For this study only female rats were used, to minimise any welfare risks due to fighting in group housed males, or mixing groups of males in the playpen. It is also important that future studies address differences between male and female rats, however this may need modifications to the enrichment protocol to minimise fighting between males in playpens.

To further refine these methods, additional MRI sequences, additional analysis methods, or post-mortem examinations beyond the scope of this study can be added to the protocol in future work. This may include ASL fMRI, in order to quantify age effects on stimulus-induced CBF changes with age, rather than using the more abstract % change in BOLD signal. Additionally, refinements to sequences could include longer angiography scans in order to quantify structural properties of the vasculature, as opposed to using a short angiography scan to aid in labelling plane positioning for ASL. Parameters to structural images could also be refined in order to give better grey/white matter contrast for calculating partial volume effects (Dukart and Berolino, 2014). As fMRI sequences are further refined, multiparametric studies can also be used to perform biophysical modelling in order to better understand how each parameter reflects changes in neurovascular coupling, which has previously been used in optical image spectroscopy studies (Dubeau et al., 2011b). Ex-vivo studies, such as electrophysiology or immunostaining, would also be beneficial to understand the cellular and molecular components of the mechanisms described.

Combining results from all MRI techniques, the reduction in size of the active BOLD region, shift to a negative glutamate change, while CBF and NAA levels remained constant, offers an explanation of how neurovascular coupling may change with age. It is possible that the reduced glutamate turnover is the cause of the observed reduction in size of the BOLD signal, without significant neuronal death or impairment of cerebral autoregulation. These methods have application in the study of neurodegenerative, neurological and vascular diseases and may enable longitudinal preclinical studies, which are relevant for progressive diseases such as stroke or Alzheimer’s disease to improve the translational relevance of such preclinical models (Sauter et al., 2002; Dijkhuizen et al., 2003; Hu et al., 2004; Sanganahalli et al., 2013). In addition, the combination of a multi-parametric fMRI protocol may enable factors influencing neurovascular coupling in response to aging and neurodegenerative conditions to be further explored. These methods also represent a refinement to animal welfare, in that animals can be kept healthy for 18 months, and animal numbers can also be reduced, which will benefit animal research as a whole.

## Data availability statement

We will upload data in spreadsheet form plus raw images/spectra to Mendeley Data on Elsevier website.

## Funding

A.C. is supported by 10.13039/501100000265Medical Research Council, United Kindgom, IMPACT DTP PhD Studentship (grant number MR/N013913/1).

## CRediT authorship contribution statement

**Andrew Crofts:** Conceptualization, Methodology, Data curation, Writing - original draft. **Melissa Trotman-Lucas:** Methodology, Supervision, Writing - review & editing. **Justyna Janus:** Methodology, Supervision, Writing - review & editing. **Michael Kelly:** Conceptualization, Funding acquisition, Methodology, Writing - review & editing. **Claire L. Gibson:** Conceptualization, Funding acquisition, Methodology, Writing - review & editing.
